# Sensitivity to scale of willingness‐to‐pay within the context of menorrhagia

**DOI:** 10.1111/hex.12452

**Published:** 2016-02-23

**Authors:** Sabina Sanghera, Emma Frew, Janesh Kumar Gupta, Joe Kai, Tracy Elizabeth Roberts

**Affiliations:** ^1^ Health Economics UnitUniversity of BirminghamBirminghamUK; ^2^ School of Clinical and Experimental MedicineUniversity of BirminghamBirminghamUK; ^3^ Division of Primary Care & National Institute for Health ResearchUniversity of NottinghamNottinghamUK; ^4^ Present address: Health Economics Research GroupBrunel University LondonUxbridgeUB8 3PHUK

**Keywords:** willingness‐to‐pay, menorrhagia multi‐attribute scale, convergent validity, EQ‐5D, sensitivity to scale

## Abstract

**Objectives:**

Willingness‐to‐pay (WTP) provides a broad assessment of well‐being, capturing benefits beyond health. However, the validity of the approach has been questioned and the evidence relating to the sensitivity of WTP to changes in health status is mixed. Using menorrhagia (heavy menstrual bleeding) as a case study, this exploratory study assesses the sensitivity to scale of WTP to change in health status as measured by a condition‐specific measure, MMAS, which includes both health and non‐health benefits. The relationship between EQ‐5D and change in health status is also assessed.

**Methods:**

Baseline EQ‐5D and MMAS values were collected from women taking part in a randomized controlled trial for pharmaceutical treatment of menorrhagia. Following treatment, these measures were administered along with a WTP exercise. The relationship between the measures was assessed using Spearman's correlation analysis, and the sensitivity to scale of WTP was measured by identifying differences in WTP alongside differences in MMAS and EQ5D values.

**Results:**

Our exploratory findings indicated that WTP, and not EQ‐5D, was significantly positively correlated with change in MMAS, providing some evidence for convergent validity. These findings suggest that WTP is capturing the non‐health benefits within the MMAS measure. Mean WTP also increased with percentage improvements in MMAS, suggesting sensitivity to scale.

**Conclusion:**

When compared to quality of life measured using the condition‐specific MMAS measure, the convergent validity and sensitivity to scale of WTP is indicated. The findings suggest that WTP is more sensitive to change in MMAS, than with EQ‐5D.

## Introduction

Contingent valuation is a method for assigning monetary values to non‐market goods, such as health‐care interventions, for use as an outcome measure within cost–benefit analysis (CBA). Willingness‐to‐pay (WTP) is the most commonly used contingent valuation approach and provides an overall measure of strength of preference expressed in monetary terms. Using this approach, individuals are asked to consider hypothetical scenarios that describe both the process and outcome of the health‐care intervention and asked to state maximum WTP for the health care good being valued. Sample average WTP values are typically used as an indication of strength of preference and can be directly compared to assess the value of alternative health‐care interventions. In a similar way to how EQ‐5D is used to inform the outcome measure for cost–utility analysis (CUA), WTP can also be used to inform the outcome for a CBA. Used in this way, it becomes a generic measure of value for treatment or services and is thus weighed against cost to measure overall cost‐effectiveness. Depending on the ratio between the incremental difference in costs and benefits (WTP) of alternative treatment options, judgements can then be made by decision makers on whether to recommend the treatment/service based on this ratio. WTP encapsulates both health and non‐health aspects of well‐being, the advantages of such an approach are ever more recognized, particularly because the National Institute for Health and Care Excellence are now also commissioning across public health and social care.^1^ Despite this, there are key limitations to the WTP approach including the difficulty with contemplating the hypothetical survey scenario, and the lack of well‐defined preferences when individuals are unfamiliar with the goods they are asked to value. The literature also refers to evidence on strategic bias and questions about the validity of the approach.^2^

The objective of this exploratory study was to examine the validity of the WTP approach using treatment for heavy menstrual bleeding (clinically termed menorrhagia) as a case example. Menorrhagia is a chronic condition with episodic symptoms which is known to affect both health and non‐health aspects of life. Generic quality of life measures, such as EQ‐5D and SF‐6D, that are focused on health alone are recommended for use in health care to be used to capture the impact of conditions. The advantage of these generic measures is that they enable comparison of effectiveness across different treatment conditions as the outcomes are measured using one commensurate unit. An interesting feature of menorrhagia is the condition's chronic but episodic nature as the symptoms occur for approximately 1 week every month which has implications for the timing of assessment when using generic measures with standard recall periods.^3^ In terms of validity, within the context of menorrhagia the condition‐specific quality of life measure, MMAS (menorrhagia multi‐attribute scale described in detail below), is considered to be the gold standard measure.^4^ Whilst condition‐specific measures can be more sensitive than generic measures, their use in decision making is limited due to the lack of comparability across conditions. There are several types of validity that one can assess including content validity *(whether all relevant aspects of the condition are considered in the instrument)*, construct validity *(determines whether an underlying relationship exists between questions in the instrument and an attribute that is measured)*, and criterion validity (*whether one attribute or set of attributes predicts an outcome based on information from other attributes*).^5^, ^6^ The focus of this paper is construct validity, or more specifically convergent validity which is the degree to which two theoretically equivalent measures converge, and within the context of contingent valuation, it is often referred to as sensitivity to scale or scope.^7^

This question is of theoretical relevance as many studies have shown that WTP demonstrates theoretical validity with WTP increasing with income,^8^ and others have focused on convergent validity with other preference elicitation measures such as time trade‐off (TTO) and standard gamble (SG).^9^ The evidence on convergent validity within the health‐care sector and sensitivity to the size of the benefit is mixed 9, 10 and in particular, the evidence relating to the sensitivity of WTP to changes in health status 9 is far from conclusive. In theory, the respondent's perception of the value of a treatment should be sensitive to changes in the size of the benefit derived from the treatment. For example, WTP demonstrates convergent validity if the WTP increases with a perception of greater improvement in treatment benefit. This study presented a unique opportunity to assess the sensitivity of WTP longitudinally as both EQ5D and MMAS data were collected at different time points, along with WTP. The sensitivity to scale of WTP can therefore be assessed in relation to EQ5D and MMAS over time.

First, we assessed the change in condition‐specific quality of life following treatment for menorrhagia, as measured by MMAS, against the WTP for this change in outcome, and second, we assessed the underlying relationship between WTP and general health‐related quality of life when measured using EQ‐5D.

## Methods

We carried out the exploratory study with women who were already participating in the NIHR funded ECLIPSE trial (ISRCTN86566246).^11^ Ethical approval was obtained from the National Research Ethics Service Committee South West‐Exeter and clinical trial authorization from the Medicines and Healthcare Regulatory Authority. Written consent was obtained from the participants.

### Study population

The ECLIPSE trial is a pragmatic, multicentre, randomized trial, comparing the clinical and cost‐effectiveness of levonorgestrel‐releasing intrauterine device (LNG‐IUS) against usual medical treatment in the primary care setting.^11^, ^12^ Women between 25 and 50 years of age that presented to their general practitioner (GP) with menorrhagia, occurring over at least three consecutive cycles, were randomized to a treatment group by telephone or web‐based central randomization service. Women were excluded if they intended to become pregnant over the next 5 years, were taking hormone replacement therapy or tamoxifen, had intermenstrual or post‐coital bleeding or examination suggestive of fibroids (abdominally palpable uterus equivalent in size to 10–12 weeks of gestation) or other pathologies, or had contraindications to, or a preference for, LNG‐IUS or usual medical treatments. The pharmaceutical treatments were either the LNG‐IUS (termed Mirena in the questionnaire) or usual medical treatment (oral treatment), which can include tranexamic acid, mefenamic acid, combined oral contraceptive or Depo‐Provera.

### Data collection

Data were collected at baseline and once symptoms had stabilized with treatment (‘post‐initial treatment effectiveness’). As the study is nested within the ECLIPSE trial, ECLIPSE trial data collection forms were used to collect baseline data. For the post‐initial treatment effectiveness data collection and for the purposes of this study, we adapted the ECLIPSE trial forms by adding an additional WTP question. Both questionnaires were sent to women in the ECLIPSE trial for completion.

#### ECLIPSE trial baseline questionnaire

As part of the trial, follow‐up women were asked to complete the condition‐specific measure MMAS and the generic health‐related quality of life measure EQ‐5D‐3L. The instruments had the following properties:

1. MMAS. MMAS is a self‐report questionnaire that has six attributes including ‘practical difficulties’, ‘social life’, ‘psychological health’, ‘physical health and well‐being’, ‘work/daily routine’ and ‘family life/relationships’. Each attribute has four levels ranging from unaffected to severely affected. For example, the wording for social life relating to severely affected reads ‘My social life is devastated during my cycle. I am unable to make any plans’. The questions refer to ‘during my cycle’, and the respondent ticks the level that most accurately reflects their experience. The measure is scored on a 0–100 scale, with 0 being worst possible state for the condition and 100 being best possible state. Each attribute has been weighted according to the menorrhagia patients’ preferences using 21 counters, which are considered to be importance points. The visual analogue scale (0–100) is then used to weight the relative importance of the levels within the attribute. The weighting for the levels is then multiplied by the weighting for the attribute. The overall score is then derived by summing the value of the levels ticked by the respondent to provide an overall score between 0 and 100.^13^
2. EQ‐5D‐3L. EQ‐5D is a generic measure of health outcome that can be used across a range of conditions. Its five attributes include ‘mobility’, ‘self‐care’, ‘usual activities’, ‘pain/discomfort’ and ‘Anxiety/depression’ The attributes each have three levels and the questions are asked with reference to ‘health today’. Responses to the instrument can be used to generate a health‐related quality of life score referred to as a ‘utility’ value expressed on a scale where 0 represents death, values below zero worse than death and 1 indicates full health.

#### Post‐initial treatment effectiveness questionnaire

The questionnaire booklets that were designed for the purpose of this study were posted in August 2012 to all eligible women in the ECLIPSE trial. This time point captured women who were either 2 or 5 years post‐initial treatment effectiveness, due to the 3‐year time period for recruitment into the trial. By post, women received (1) a patient information sheet outlining the purpose of the work and; (2) an ‘ex‐post’ (post‐initial treatment effectiveness) questionnaire; and (3) a prepaid stamped addressed envelope to return the completed questionnaire.

The objective of the ex‐post questionnaire was to elicit a WTP value for the pharmaceutical treatment that the women were currently taking, either LNG‐IUS or oral treatment. In this context therefore, average maximum WTP values were derived *after* the change in outcome had occurred, from respondents who had experience of the condition and experience of the treatment. Hence, WTP was elicited from the ex‐post perspective. The WTP value therefore reflected the direction and level of change in outcome over time in response to treatment. It is a commonly practiced approach to consider use value when eliciting WTP in health care.^14^, ^15^, ^16^

Maximum WTP values were elicited for both LNG‐IUS and oral treatment using the self‐complete ex‐post booklet questionnaire. The booklet was similar in design to the trial questionnaire and captured data on condition‐specific quality of life (MMAS), WTP, socio‐demographic details and health‐related quality of life using the EQ‐5D‐3L.

The MMAS was first presented in the questionnaire, followed by questions to determine current and previous treatment taken as part of the ECLIPSE trial. Respondents were asked for their maximum monthly out of pocket WTP value for their *current* treatment. The time frame of payment of ‘up until menopause’ was explicitly stated to ensure WTP values were not overestimated.^17^ To elicit WTP, the payment scale elicitation format was used as it has a higher completion rate than other methods that can be used in a postal questionnaire.^18^ The scale range used was £0–£500, and an open‐ended option for values greater than £500 was offered. To assess the validity of the WTP responses and the respondents understanding of the WTP question, we then asked the respondent to outline reasons for their WTP values in an open‐ended question. This approach enabled an assessment of responses as well as providing insight into the way in which the WTP question was interpreted. To ensure the WTP values were realistic, that is within the respondent's resources, and in line with good practice, a reminder was included asking the women to consider the amount that they can actually afford to pay.^19^

### Analysis

For the analysis, baseline data were taken from the ECLIPSE trial questionnaire and post‐initial treatment effectiveness data were from the purposely developed WTP questionnaire. Scores were calculated for the baseline MMAS, obtained from the ECLIPSE trial, and the current (‘ex‐post’) MMAS score. Similarly, baseline and follow‐up EQ‐5D quality of life score was calculated for every woman.^20^ Descriptive statistics are reported for each measure and a paired *t*‐test was conducted to determine the difference between follow‐up and baseline values at the 5% level. Cohen's effect size (mean change divided by standard deviation) is calculated for each of the measures where 0.2–0.5 indicates a small change, 0.5–0.8 moderate and >0.8 large.^21^ As the assessment of the validity of WTP is the aim of this study, and not the incremental difference between treatment arms, the WTP values for both treatments were combined. The CUA and CBA which consider the treatment effect by group are presented elsewhere.^12^, ^22^ Thus, the WTP for overall treatment (both LNG‐IUS and oral treatment) for menorrhagia is used to assess the convergent validity. The association between WTP and change in condition‐specific quality of life (MMAS), from baseline to the ex‐post values, was first assessed. Second, the association between WTP and change in general health‐related quality of life as measured by the EQ‐5D was assessed. Finally, the association of change in MMAS and change in EQ‐5D was explored. The associations between the measures were assessed by Spearman's correlation analysis. A Rho value between 0.10 and 0.29 indicates a small association, 0.30–0.49 medium association and greater than 0.5 a large association.^23^ The percentage improvement in MMAS from baseline to current time point was also calculated, using percentage improvements (<25, 26–50, 51–75 and >75%) to establish the extent to which WTP increased with improvement in MMAS from baseline. The Wilcoxon rank–sum test was carried out to identify whether the differences between the WTP values for each percentage change were significant at the 5% level. The qualitative reasons for the WTP values were further analysed and categories generated based on a previous published WTP study.^14^ The qualitative information was supplemented by exploring the influence of prominent numbers on the WTP results. Prominent numbers are those that are typically selected by respondents and include £0, 1, 2, 5, 10, 20, 50, 100, 200, 500 and so on.^24^ The selection of prominent numbers is thought to be related to the respondent's perception of the difficulty of the task and their knowledge of the intervention.^25^ Where appropriate, GBP is converted to USD using a currency conversion website www.fxexchangerate.com; (£1 = US$0.665; 2013). We carried out all data analyses in STATA (v11.0) and Microsoft Excel.

## Results

All of the two hundred and seventy‐two women who were eligible to complete the questionnaire in the trial received a copy. One hundred and sixty‐three questionnaires (60%) were received; however, 78 of these questionnaires were excluded as the women were no longer taking either of the randomized pharmaceutical treatments, and therefore, the WTP section was not applicable to these women. Our intention was to obtain the value for either treatment (LNG‐IUS or oral treatment) and the question posed in the questionnaire referred to ‘current treatment’. As these women were either no longer taking any treatment due to menopause or other reasons, it would not be appropriate to use values for ‘current treatment’. However, the values of those who have crossed over to the other treatment are included to ensure that the values of those who were unhappy with their original treatment were included.

Of the remaining 85 women who returned the questionnaire and were currently taking one of the randomized treatments, 4 (4%) women did not provide a WTP value and MMAS score for their current treatment, and 11 (13%) protest answers were identified from the qualitative explanations offered for the WTP value. Protest responses are defined as an explicit objection to being asked to ‘pay’ for health care and therefore a misunderstanding of the hypothetical nature of the exercise and are therefore invalid. These 15 non‐responses and protest answers were removed from the analysis. It is generally accepted in the literature that qualitative information associated with the protest responses should be assessed to determine whether the values are genuine WTP values or whether the respondent is protesting against the exercise. There is a debate in the literature about the inclusion of protest responses.^14^ Where protests are identified, it has been argued that these should not be included in the analysis as they are not valid responses; however, some authors have expressed concerns about their exclusion if the characteristics of the excluded and included group are not assessed.^14^ There were no significant differences between the characteristics of this excluded group to the sample analysed (see online supplementary material). In total, 70 respondents gave a WTP value for either LNG‐IUS or oral treatment and the characteristics of this sample are presented in Table 1. The proportion of respondents that had a household income of less than £30 000 (US$45 113) was approximately 50%, which was lower than the national average where 65% are below approximately £27 000 (US$40 602).^26^

**Table 1:** Sample characteristics of WTP respondents

Variable	Treatment (*n* = 70)
Expected age of menopause (yrs) [SD]	53.80 [2.50]
Age [SD]	48.09 [3.93]
Marital status (%)	
Married or living with partner	53 (76)
Not	17 (24)
Employment status (%)	
Employed (FT)/(PT)	56 (80)
Not	14 (20)
Household income (%)	
Less than £20 000 (<$30 075)	22 (32)
£20 001–30 000 ($30 077–$45 113)	14 (20)
£30 001–40 000 ($45 114–$60 150)	10 (14)
£40 001–50 000 ($60 152–$75 188)	9 (13)
More than £50 000 (>$75 189)	13 (20)
Main earner (%)	
Yes	32 (46)
No	37 (54)

FT, full‐time; PT, part‐time; SD, standard deviation. (US$).

### Treatment response

The average maximum WTP for treatment for menorrhagia was approximately £27 (US$41) per month (see Table 2). The average combined MMAS score for treatment doubled from approximately 43 at pre‐treatment to 85 following treatment, generating a statistically significant improvement in health status as measured by this instrument (*P* = 0.000). According to the Cohen's standardized effect size, the mean effect of 1.97 observed on MMAS would indicate a large change as the effect size is greater than 0.8. Whilst average EQ‐5D values increased significantly (*P* = 0.0168) from 0.789 pre‐treatment to 0.880 post‐initial treatment effectiveness, the mean effect size of 0.36 indicates a small change using the same Cohen's criteria (Table 3). Whilst 0.789 pre‐treatment to 0.880 post‐initial treatment effectiveness generates a mean effect size of 0.36 which would indicate a small change using the same Cohen's criteria, it should be noted that this level of change in EQ‐5D would lead to a change in one level of one attribute; for example, using the pain attribute a change would be observed from some pain to no pain which could be deemed clinically important.

**Table 2:** Descriptive statistics for WTP

	Valid responses	Mean (Median) [$]	Min–Max [$]	SD [$]
WTP	70	£26.99 (£10) [$40.59 ($15)]	£0–£500 [$0–$752]	£60.73 [$91.32]

SD, standard deviation; WTP, willingness‐to‐pay. (US$).

**Table 3:** Average scores for the instruments

	Mean MMAS [SD]	EQ‐5D [SD]	WTP ($)
Baseline (*n* = 70)	43.23[21.22]	0.789 [0.250]	
Post‐initial treatment effectiveness (*n* = 70)	85.00 [23.15]	0.880 [0.215]	£26.99 ($40.59)
Change (*n* = 70)	41.77 [32.03]	0.09 [0.31]	

MMAS, Menorrhagia multi‐attribute scale; SD, standard deviation; WTP, willingness‐to‐pay.

### Respondent's understanding of WTP

**Figure 1:**
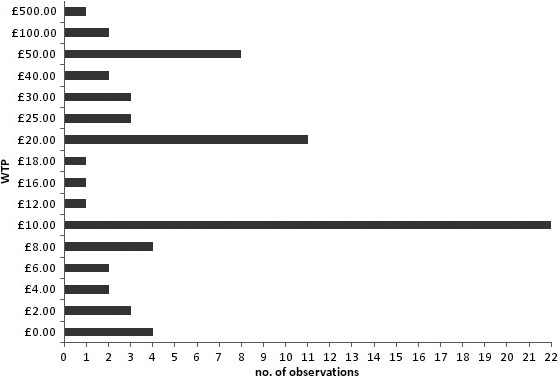
Frequency of WTP values.

Among the 70 women who provided a WTP value, 69 provided a reason for the value. Nine categories of reasons for a WTP value were generated from the qualitative information from all of the women, which included protests and non‐response. The categories of reasons for the sample analysed are presented in Table 4. It can be seen that for both LNG‐IUS and oral treatment that ‘R4: affordability’ and ‘R7: effects of treatment’ are the most commonly cited reason for a WTP value. In addition to this finding, it can be seen from Fig. 1, which presents the WTP values for treatment against the number of observations, that the most commonly selected WTP values were prominent numbers, those are £10 and £20, which make up 31 and 16% of the sample which could indicate difficulty with the WTP exercise or a tendency to round numbers (discussed later).

**Table 4:** Explanation given for WTP value (sample analysed, ex‐post)

Category	Explanation	Total *n* (%)
R2	Subject expressed difficulty estimating WTP owing to: Difficult to answer Cannot put a price on health care	8 (7)
R3	WTP based on nominal amount Arbitrary sum/guess/out of thin air	1 (0.9)
R4	WTP reflects ability to pay (affordability) Maximum affordable amount given current situation	37 (35)
R5	WTP reflects reasonable value NHS should pay but this is a reasonable limit	8 (7)
R6	WTP reflects cost of treatment Attempted to estimate cost Used a comparator such as prescription costs	5 (5)
R7	WTP reflects effect of treatment In terms of effectiveness outcomes In terms of process utility	30 (28)
R9	Related to cost of sanitary wear Washing clothes/wipes/painkillers	13 (12)
R10	Misunderstood exercise but provided WTP value	5 (5)
Total		107^a^

NHS; National Health Service, WTP; willingness‐to‐pay.

a Some respondents gave more than one reason for their WTP value. Categories R1 and R8 were related to protest responses and are deleted from the table as they were not included in the sample analysed.

### Associations

Average maximum WTP was significantly positively correlated with change in MMAS (*P* = 0.025) (Table 5). That is, the greater the change in health state, as measured by change in MMAS, the greater the WTP value. However, the strength of the relationship is relatively small, generating a rho value of 0.27. In contrast, the association between change in EQ‐5D and change in MMAS did not show statistical significance (*P* = 0.059), despite demonstrating a positive correlation (r = 0.23). An unusual, though non‐significant result is generated in the association between WTP and change in EQ‐5D, as a minor negative correlation (r = −0.02) is observed.

**Table 5:** Associations between measures

	Change in MMAS (rho)	WTP (rho)	Change in EQ‐5D (rho)
Change in MMAS	1.0000		
WTP	0.2674^a^	1.000	
Change in EQ‐5D	0.2265	−0.0158	1.000

a *P* = 0.0252 (<0.05) MMAS; menorrhagia multi‐attribute scale, WTP; willingness‐to‐pay.

When WTP for percentage change in MMAS is assessed, the mean WTP increases as the change in health status increases (from approximately £16 to £63 (US$25–US$95)). Whilst the mean values would suggest that WTP would continue to increase the greater the change in health status, the median values illustrate the skewness of the data but still demonstrate an increase in WTP as health status improves (Table 6). The significance tests show the WTP values between ‘<25%’ and ‘51–75%’ to be significantly increased as percentage change in MMAS increases (*P* = 0.033; *P* < 0.05).

**Table 6:** Mean WTP against percentage improvement in MMAS

% change in MMAS	Mean MMAS [SD]	No. obs	Mean WTP [SD] ($)	Median ($)	Min–Max WTP ($)
<25	1.27 [12.82]	21	£16.29 [£17.97] ($24.50)	£10 ($15)	£0–£50 ($0–75)
26–50	36.13 [8.3]	14	£20.86 [£24.65] ($31.37)	£10 ($15)	£6–£100 ($9–$150)
51–75	60.95 [9.04]	24	£23.38 [£20.63] ($35.16)	£20 ($30)	£8–£100 ($5–$150)
>75	84.44 [10.00]	11	£63.09 [£145.86] ($94.87)	£20 ($30)	£0–£500 ($0–$752)

MMAS; menorrhagia multi‐attribute scale, SD, standard deviation; WTP; willingness‐to‐pay. (US$).

## Discussion

In this exploratory study, we aimed to assess the convergent validity of WTP within the context of menorrhagia by comparing change in the condition‐specific measure (MMAS) to (1) change in WTP and (2) the generic health‐related quality of life measure, EQ‐5D‐3L. Overall, our findings suggest the convergent validity of WTP as it behaves as would intuitvely be expected in response to change in health status, as measured by MMAS. Specifically, following treatment, WTP increases with a greater improvement in treatment benefit, and the correlation between the change in condition‐specific MMAS and WTP suggests statistical significance.

An association between MMAS and the generic measure, EQ‐5D, was not observed as the change in EQ‐5D scores before and after treatment was not significantly associated with the equivalent change in MMAS. This result suggests that WTP is more sensitive to change in the condition‐specific measure (MMAS), than the generic measure (EQ‐5D). We hypothesize that this finding is attributed to the EQ‐5D instrument being designed as a generic health‐related quality of life measure, which is not focussed specifically on menorrhagia. We suggest that WTP is more sensitive given that it has the potential to measure both health and non‐health aspects of quality of life that are important to women who suffer with menorrhagia, which are also encompassed by the MMAS measure.

Qualitative reasons for the WTP responses provided a further opportunity to assess the reliability of the WTP values. The analyses of the reasons confirmed that respondents were considering the ‘value’ of the treatment as was theoretically expected. These values are therefore reflective of what the theory says people consider when completing WTP exercises. However, the selection of prominent numbers could be related to the perceived difficulty of the task 25 where respondents provide less precise values when they do not feel they have adequate knowledge of the good being valued.^24^ In this study, the WTP values were elicited from the ex‐post perspective where respondents have experience of, and are knowledgeable about, the treatments and despite this, prominent numbers were most commonly selected. This indicates that although respondents stated ‘true’ WTP values as confirmed by the qualitative responses, the selection of prominent numbers alludes to the WTP elicitation task being difficult to complete.

Finally, as there are several possible approaches for eliciting WTP, the method used in the analysis reported here requires some justification. With respect to the WTP question, the time period ‘up until menopause’ seemed intuitive given that menorrhagia ceases at menopause and the scale of £0–£500 was thought most suitable, given that the questionnaire asked respondents to provide a monthly WTP value. The monthly payment time frame was used because women generally pay monthly (or every 3 months) for prescriptions for menorrhagia, for sanitary protection and they experience the benefits of treatment on a monthly basis. The out of pocket payment vehicle was deemed appropriate for this context because whilst the full price for treatment is not paid in the UK, patients do make an out of pocket payment for prescriptions for oral treatment in the UK. Although this private payment does not exist for LNG‐IUS, the existence of private payment within this context is likely to minimize the issue of hypothetical bias.

To our knowledge, this is the first study to assess the convergent validity of WTP against a change in condition‐specific quality of life measure in menorrhagia. It is also the first to compare the correlation of WTP and change in EQ‐5D with a condition‐specific quality of life measure.

A limitation of this exploratory study is the sample size used. Given that the significant difference was observed between the two percentage change categories with the greatest number of observations, it is likely that the remaining categories were not found to be significant due to the limited sample size for the groups ‘26–50%’ and ‘>75%’. Thus, there may not have been sufficient power to detect a significant difference between these groups. The findings from this exploratory study, however, can be used to inform future sample size calculations for subsequent WTP studies in the area. Prior to this study, it was not readily possible to calculate sample size requirements as *a priori* data on the distribution of WTP values for the LNG‐IUS or oral treatment were not available.^27^ This study therefore enables researchers to identify the number and range of responses given to determine how many respondents are required to detect a certain difference in WTP across treatments, that is the WTP value that constitutes a meaningful difference in improvements from baseline or between treatments.

To our knowledge, no other study has assessed the sensitivity to scale of WTP according to a longitudinal change in outcome measured by a condition‐specific measure. Other related studies have, however, assessed the relative sensitivity of WTP and time trade‐off (TTO) for changes in described dimensions of health states and have tentatively suggested that WTP is sensitive to change in different levels of health within the same dimension.^9^ The sensitivity of (*ex ante*) WTP with a condition‐specific measure at one time point has also been explored.^28^, ^29^ Radtke *et al*.^28^ assessed the relationship between WTP, EQ‐5D and a condition‐specific measure for vitiligo; and Schiffner *et al*.^29^ assessed the sensitivity of WTP and TTO with a condition‐specific measure for psoriasis. Baseline EQ‐5D (or TTO) and condition‐specific scores for current health state were identified, and hypothetical WTP values were elicited for a cure. Both studies found that WTP had a significant correlation with the condition‐specific measure, and Radtke *et al*.^28^ also showed WTP and EQ‐5D to have negative correlation.

Other psychometric properties of WTP and EQ‐5D in menorrhagia have also been explored. A review of psychometric properties of measures used in menorrhagia^3^ identified four key studies. One study assessed the consistency of responses of WTP from women with menorrhagia and found a lack of external reliability of WTP.^30^ The second found EQ‐5D to be unsuitable for patients with menorrhagia.^4^ In the third, MMAS was statistically associated with changes in satisfaction post‐initial treatment effectiveness, whilst EQ‐5D was not.^31^ Finally, a lack of sensitivity of EQ‐5D to changes in quality of life associated with menorrhagia was observed.^32^

Further research is required to consolidate our findings using a larger sample size, the requirements for which can now be derived based on our study. Additional research could explore the convergent validity of other methods of valuation such as SF‐6D in such a condition, assessing changes in SF‐6D in relation to WTP and MMAS to determine the extent to which SF‐6D reflects changes in MMAS. This exploratory study suggests that there is the potential, and a benefit, to the use of WTP in chronic conditions with episodic symptoms which impact on health and non‐health aspects of life.

## Conflicts of interest

The authors declare that they have no conflict of interest.

## Source of funding

This study research relates to a PhD studentship which was funded by the National Institute for Health Research Health Technology Assessment programme, UK.

## Supporting information


**Table S1.** Characteristics of sample analysed and excluded respondents
