# Supplementary material for: Sensitivity to scale of willingness‐to‐pay within the context of menorrhagia
Source: Health Expect. 2016 Feb 23;20(2):232–42. doi: 10.1111/hex.12452 (PMC5354011; doi:10.1111/hex.12452)
Supplement: Supplementary file 1 — Table S1. Characteristics of sample analysed and excluded respondents [file HEX-20-232-s001.docx]

**Supplementary online material**

Table S1. Characteristics of sample analysed and excluded respondents

| **Variable** | **Included (n=70)** | **Excluded (n=15)** |
| --- | --- | --- |
| **Expected age of menopause (yrs) [SD]** | 53.80 [2.50] | 55 |
| **Age [SD]** | 48.09 [3.93] | 46.9 [4.63] |
| **Marital status (%)** |  |  |
| Married or living with partner | 53 (76%) | 12 (80%) |
| Not | 17 (24%) | 3 (20%) |
| **Employment status (%)** |  |  |
| Employed (FT)/(PT) | 56 (80%) | 12 (86%) |
| Not | 14 (20%) | 2 (14%) |
| **Household income (%)** |  |  |
| Less than 20,000 (<$30,075) | 22 (32%) | 3 (25%) |
| 20,001-30,000 ($30,077 - $45,113) | 14 (20%) | 2 (17%) |
| 30,001-40,000 ($45,114 - $60,150) | 10 (14%) | 1 (8%) |
| 40,001-50,000 ($60,152 - $75,188) | 9 (13%) | 1 (8%) |
| More than 50,000 (>$75,189) | 13 (20%) | 5 (42%) |
| **Main earner (%)** |  |  |
| Yes | 32 (46%) | 6 (43%) |
| No | 37 (54%) | 8 (57%) |

The Fishers Exact test was carried out for categorical data and the Kruskal Wallis test for continuous data to determine whether the excluded protest group was significantly different to the sample included in the analysis. A significant difference was not observed between the included and excluded group for marital status (p=1.00), employment status (p=1.00), income (p=0.674), main earner (p=1.00) or patient age (p=0.714).
